# Exit Rates of Accountable Care Organizations That Serve High Proportions of Beneficiaries of Racial and Ethnic Minority Groups

**DOI:** 10.1001/jamahealthforum.2022.3398

**Published:** 2022-09-30

**Authors:** Sunny C. Lin, Karen E. Joynt Maddox, Andrew M. Ryan, Nicholas Moloci, Addison Shay, John Malcolm Hollingsworth

**Affiliations:** 1 Division of General Medical Sciences, Department of Medicine, Washington University School of Medicine in St. Louis, St Louis, Missouri; 2 Institute for Informatics, Washington University in St. Louis, St Louis, Missouri; 3 Institute for Public Health, Washington University in St. Louis, St Louis, Missouri; 4 Cardiovascular Division, Department of Medicine, Washington University School of Medicine in St. Louis, St Louis, Missouri; 5 Health Management and Policy, University of Michigan School of Public Health, Ann Arbor; 6 Department of Health Policy and Management, University of North Carolina, Chapel Hill; 7 Dow Division of Health Services Research, Department of Urology, University of Michigan, Ann Arbor

## Abstract

**Importance:**

The Medicare Shared Savings Program provides financial incentives for accountable care organizations (ACOs) to reduce costs of care. The structure of the shared savings program may not adequately adjust for challenges associated with caring for patients with high medical complexity and social needs, a population disproportionately made up of racial and ethnic minority groups. If so, ACOs serving racial and ethnic minority groups may be more likely to exit the program, raising concerns about the equitable distribution of potential benefits from health care delivery reform efforts.

**Objective:**

To evaluate whether ACOs with a high proportion of beneficaries of racial and ethnic minority groups are more likely to exit the Medicare Shared Savings Program and identify characteristics associated with this disparity.

**Design, Setting, and Participants:**

This retrospective observational cohort study used secondary data on Medicare Shared Savings Program ACOs from January 2012 through December 2018. Bivariate and multivariate cross-sectional regression analyses were used to understand whether ACO racial and ethnic composition was associated with program exit, and how ACOs with a high proportion of beneficaries of racial and ethnic minority groups differed in characteristics associated with program exit.

**Exposures:**

Racial and ethnic composition of an ACO’s beneficiaries.

**Main Outcomes and Measures:**

Shared savings program exit before 2018.

**Results:**

The study included 589 Medicare Shared Savings Program ACOs. The ACOs in the highest quartile of proportion of beneficaries of racial and ethnic minority groups were designated high-proportion ACOs (145 [25%]), and those in the lowest 3 quartiles were designated low-proportion ACOs (444 [75%]). In unadjusted analysis, a 10–percentage point increase in the proportion of beneficiaries of racial and ethnic minority groups was associated with a 1.12-fold increase in the odds of an ACO exit (95% CI, 1.00-1.25; *P* = .04). In adjusted analysis, there were significant associations among high-proportion ACOs between characteristics such as patient comorbidities, disability, and clinician composition and a higher likelihood of exit.

**Conclusions and Relevance:**

The study results suggest that ACOs that served a higher proportion of beneficaries of racial and ethnic minority groups were more likely to exit the Medicare Shared Savings Program, partially because of serving patients with greater disease severity and complexity. These findings raise concerns about how current payment reform efforts may differentially affect racial and ethnic minority groups.

## Key Points

**Question:**

Are accountable care organizations (ACOs) that serve a high proportion of beneficaries of racial and ethnic minority groups more likely to exit the Medicare Shared Savings Program?

**Findings:**

In this cohort study of 589 Medicare Shared Savings Program ACOs, from January 2012 to December 2018, ACOs with a higher proportion of patients of racial and ethnic minority groups were more likely to exit the Medicare Shared Savings Program. In multivariable analysis, the higher exit rate was associated with significant differences in beneficiary complexity and ACO structure.

**Meaning:**

The study results suggest that ACOs that serve racial and ethnic minority groups are also more likely to serve beneficiaries with complex medical and social needs; recent changes in the payment structures of the Medicare Shared Savings Program may be associated with the exit of ACOs that serve these populations, which may be associated with racial and ethnic disparities in ACO access.

## Teaser

This cohort study examines whether accountable care organizations with a high proportion of racial and ethnic minority groups are more likely to exit the Medicare Shared Savings Program.

## Introduction

With 477 accountable care organizations (ACOs) collectively responsible for 10.7 million assigned lives, the Shared Savings Program (SSP) is Medicare’s largest ACO initiative.^1^ Organizations participating in the SSP face financial risks and rewards for meeting cost targets each year. The SSP is a voluntary program, meaning participating ACOs can exit during any year. Prior work has found that since the launch of the SSP in 2012, approximately 30% of participating ACOs have exited. Organizational factors associated with program exit included failure to earn shared savings, leadership structure, and clinician composition.^2^

However, to our knowledge, little attention has been paid to the racial and ethnic composition of ACOs that exit, particularly whether exit rates are associated with the proportion of ACO beneficiaries who are members of racial and ethnic minority groups (ie, beneficiaries in the Medicare enrollment database who self-reported as Asian, Black, Hispanic, Native American/Alaskan Native, and/or Pacific Islander). This is important, given existing long-standing disparities in health care access and outcomes among racial and ethnic minority groups.^3^ Medicare beneficiaries who are members of racial and ethnic minority groups are more likely to have more complex medical and social needs, such as multiple comorbidities, disabilities, and lower wealth, that are associated with increased barriers to care and poorer care outcomes.^4^ The principle of justice of the Belmont Report suggests that publicly funded innovations, such as the SSP, ought to include all populations that stand to benefit.^5^ Moreover, to the extent that ACOs improve care quality and cost for their aligned beneficiaries, disparities in ACO dropout rates may exacerbate existing health care–related disparities.

Yet, recent payment reform programs have not been designed with racial and ethnic minority groups in mind.^6^ Before 2017, the spending benchmarks of ACOs were based on their own historical performance. Consequently, ACOs that served a high proportion of beneficiaries of racial and ethnic minority groups had higher benchmarks and were more likely to achieve savings.^7^ However, in 2017, despite simulations suggesting that it would negatively affect ACOs that care for racial and ethnic minority groups,^7^ the US Centers for Medicare & Medicaid Services (CMS) began incorporating regional trends into SSP benchmarking calculations without considerations or adjustments for social needs. Consequently, ACOs that care for racial and ethnic minority groups were being directly compared with nearby ACOs that serve more privileged populations. Further, in 2018, CMS announced that all ACOs would be required to take on down-side risk on an accelerated timeline, placing additional pressure on SSP ACOs that care for racial and ethnic minority groups. Consequently, SSP ACOs that serve a higher proportion of racial and ethnic minority groups may have been more likely to exit.

Therefore, we conducted a retrospective observational study using national data on Medicare SSP ACOs from 2012 to 2018. This study fills a gap in our understanding of whether current SSP policies are inclusive of racial and ethnic minority groups. Results from this study may inform policies that support equitable care delivery in payment reform efforts, an important objective given CMS’s goal to have 100% of Medicare beneficiaries (which include roughly 14 million individuals of racial and ethnic minority groups)^8^ in risk-bearing models by 2030.^9^

## Methods

### Data Sources and Study Population

We combined claims data from January 1, 2012, to December 31, 2018, on a 20% random sample of Medicare beneficiaries with corresponding years of the SSP ACO Public Use File. For beneficiaries enrolled in fee-for-service Medicare, we analyzed claims from the Medicare Provider Analysis and Review, Outpatient, and Carrier Research Identifiable Files. Through the SSP Beneficiary-level Research Identifiable File, we determined which beneficiaries were assigned to an SSP ACO during a given year along with their self-reported race and ethnicity and other beneficiary characteristics. We included only beneficiaries who had continuous Parts A and B coverage in that year. The institutional review board at the University of Michigan deemed this study exempt from oversight and informed consent because all patient data were deidentified. The reporting of this study conforms to Strengthening the Reporting of Observational Studies in Epidemiology (STROBE) reporting guidelines.

We obtained ACO organizational characteristics from the SSP Provider-level Research Identifiable File and the Torch Insight database. The Torch Insight database is a validated database that contains more than 30 fields of information on ACOs and is updated regularly through public records and interviews. We obtained environmental characteristics from the Area Health Resource File based on the county of the most common zip code of an ACO’s beneficiaries.

We excluded ACOs that exited the program but re-entered later and ACOs that formed in 2018 because they did not have an opportunity to exit before the end of the study period. The final analytic data set included all ACOs that joined the SSP from 2012 to 2017 that had all examined covariates in their entry and final year of observation.

### ACO Exit

We defined ACOs that exited as those whose ACO identification number appeared in the SSP Provider-Level Research Identifiable File for at least 1 study year but did not reappear in a subsequent year. We created a binary indicator for whether an ACO exited the SSP program before the final year of the data set, 2018.

### Main Independent Variable

To identify ACOs that serve a higher proportion of beneficaries of racial and ethnic minority groups, we used data from the SSP Beneficiary-Level Research Identifiable File. Race and ethnicity were determined using the Medicare enrollment database codes that were drawn from beneficiaries’ social security data. Racial and ethnic minority groups were identified as all beneficiaries not coded as non-Hispanic White in the enrollment database. We calculated the proportion of each ACO’s beneficiaries who were members of racial and ethnic minority groups and considered high-proportion ACOs as those in the highest quartile of percentage of beneficaries of racial and ethnic minority groups in their entry year and low-proportion ACOs as all other ACOs^10,11^ (eFigure in the Supplement). We chose to group race and ethnicity minority group categories together because we were primarily interested in race and ethnicity as an ACO characteristic rather than an individual characteristic. Furthermore, prior studies have demonstrated that racial and ethnic minority groups more broadly face a host of challenges associated with power and privilege in the US.^12^ However, recognizing that collapsing racial and ethnic categories erases important differences between heterogenous populations, we also ran analyses separately for each of the 5 US census categories for race and ethnicity.

### Beneficiary, Organizational, and Environmental Characteristics

Drawing on previous literature,^2,10,13^ we included the following covariates in our analysis. Beneficiary characteristics included percentage with a disability, percentage who were dual eligible for Medicaid and Medicare, and average percentile of hierarchical condition case risk (HCC) scores for aged non–dual-eligible beneficiaries. Organizational characteristics included earned shared savings, starting year in the program (ie, cohort), number of assigned beneficiaries (in hundreds), number of clinicians (in hundreds), percentage of clinicians that were primary care clinicians, percentage of clinicians that were advanced practice clinicians, leadership structure (physician-led or hospital-led or physician-hospital partnership), and level of out-of-network care. Earned shared savings were measured as a binary variable indicating whether the ACO earned financial incentives during the year prior using the SSP ACO Public Use File. We calculated the level of out-of-network care using the percentage of total primary and specialty outpatient visits (ie, preventive visits, annual wellness visits, and other outpatient visits) delivered out of the SSP network.^13,14^

We also included the following environmental characteristics from the most common zip code of residence among an ACO’s assigned beneficiaries: urbanicity (urban, suburban, rural), percentage dual Medicaid/Medicare eligible, percentage with an income less than the poverty line, median household income, and number of primary care clinicians per 1000 residents.

### Statistical Analysis

First, we conducted bivariate analyses using 2-tailed χ^2^ tests for categorical variables and *t* tests for continuous variables to describe whether high-proportion ACOs differed in exit rates, shared savings rates, and beneficiary, organizational, or environmental characteristics from low-proportion ACOs. For exit and earned shared savings rates, we also tested to see if they varied by year.

Second, we conducted cross-sectional logistic regression analyses to determine whether an ACO’s racial and ethnic minority group composition was associated with its likelihood of SSP exit. Each model was run at the ACO level for the ACO’s exit year/last year of the study period (ie, 2018) using a continuous measure of proportion of beneficiaries of racial or ethnic minority groups as the independent variable. The first model included only the main predictor, the second model included earned shared savings, the third included beneficiary characteristics, and the fourth included organizational and environmental characteristics. We compared the effect sizes of these characteristics with those identified as significantly different between high and low-proportion ACOs to identify potential mechanisms to support the retention of high-proportion ACOs.

Finally, we used the fully adjusted model to predict marginal effects for each covariate, holding all other covariates at subgroup means/modes for high-proportion ACOs and low-proportion ACOs (ie, at levels presented in Table 1). To determine if effect sizes were significantly different between low-proportion and high-proportion ACOs, we reran the full model interacting race and ethnicity with each covariate.

**Table 1.** Characteristics of Shared Savings Program ACOs by Low and High Proportion of Beneficiaries of Racial and Ethnic Minority Groups^a^

Characteristic	Low-proportion ACOs (n = 444)	High-proportion ACOs (n = 145)	*P* value
Percentage of individuals of racial and ethnic minority groups, mean (SD)^b^	11.2 (6.4)	39.3 (15.1)	<.001
**Beneficiary characteristics**			
With disabilities, %	11.8 (5.5)	15.5 (8.6)	<.001
Dual Medicaid eligible, %	7.4 (7.0)	18.0 (17.0)	<.001
Percentile of average HCC risk score for aged non–dual-eligible beneficiaries	41.3 (27.2)	51.4 (30.7)	<.001
**Organizational characteristics**			
Program entry cohort, No. (%)			
2012	49 (11)	52 (36)	<.001
2013	75 (17)	20 (14)	
2014	81 (18)	30 (21)	
2015	77 (17)	12 (8)	
2016	85 (19)	15 (10)	
2017	77 (17)	16 (11)	
No. of beneficiaries (hundreds)	32.2 (33.7)	24.9 (29.0)	.02
Organizing entity, No. (%)			
Physician led	196 (44)	80 (55)	.048
Hospital led	88 (20)	27 (19)	
Both	160 (36)	38 (26)	
Risk model, No. (%)			
Always upside	384 (86)	119 (83)	.19
Ever downside risk	60 (14)	26 (18)	
No. of clinicians (hundreds)	8.2 (11.7)	8.6 (14.9)	.74
Primary care clinicians, %	35.8 (16.0)	39.8 (16.6)	.01
Advanced practice clinicians, %	28.0 (12.0)	25.7 (15.1)	.06
Out-of-network care, %	47.3 (16.0)	53.8 (16.5)	<.001
**Community characteristics**			
Geography, No. (%)			
Suburban	210 (48)	31 (21)	<.001
Urban	234 (53)	114 (79)	
Dual Medicaid/Medicare eligible, %	1.3 (2.9)	0.4 (0.5)	.002
Income level less than the poverty line, %	13.5 (4.7)	14.8 (5.7)	.01
No. of PCPs per 1000 residents	0.93 (0.40)	0.90 (0.30)	.36
County median household income (in $1000)	65.1 (19.6)	67.1 (20.9)	.30

Abbreviations: ACOs, accountable care organizations; HCC, hierarchical condition case; PCP, primary care physician.

^a^ The ACOs in the highest quartile of proportion of beneficaries of racial and ethnic minority groups were designated high-proportion ACOs, and ACOs in the lowest 3 quartiles were designated low-proportion ACOs.

^b^ Unless otherwise specified, number represents means (SD).

### Sensitivity Analyses

To examine the sensitivity of the findings to exogenous events that may have occurred during any single study year, we reran the unadjusted bivariate regression model, dropping each year, and compared effect sizes across models. To test the sensitivity of the findings to the specification of “racial and ethnic minority groups,” we ran 3 additional sensitivity analyses. First, we repeated the main analyses using separate variables for each census category of race and ethnicity (ie, Asian/Pacific Islander, Black, Hispanic, Native American/Alaskan Native, and Other). We also repeated the analyses using the imputed race code provided by the Research Triangle Institute, which attempts to enhance the accuracy of race and ethnicity codes by identifying Asian and Hispanic beneficiaries based on first or last name.^15^ Finally, we repeated the analyses, replacing the continuous measure of race and ethnicity with the binary measure for high-proportion ACOs.

Effects were considered statistically significant at *P* < .05. All analyses were completed in Stata, version 15.1 (StataCorp).

## Results

### Study Population

The study population included 589 SSP ACOs. The ACOs in the highest quartile of proportion of beneficaries of racial and ethnic minority groups were designated high-proportion ACOs (145 [25%]; percentage of beneficiaries of racial and ethnic minority groups ranged from 25.6% to 94.0%), and ACOs in the lowest 3 quartiles were designated low-proportion ACOs (444 [75%]; percentage of beneficiaries of racial and ethnic minority groups ranged from 1.5% to 25.5% [eFigure in the Supplement]). Compared with low-proportion ACOs, high-proportion ACOs served a significantly higher proportion of beneficiaries with disabilities, dual Medicaid-eligible beneficiaries, and beneficiaries with a higher average HCC risk score. They were also more likely to have joined the SSP in an earlier cohort, be physician led, have a higher proportion of primary care clinicians, have higher levels of out-of-network care, be located in urban environments, serve communities with a lower proportion of dual Medicaid-eligible residents, and have a higher proportion of people with an income level less than the poverty line (Table 1).

### Likelihood of Earning Shared Savings and Program Exit

Between 2012 and 2018, 55 high-proportion ACOs (40%) earned shared savings in their penultimate year compared with 117 low-proportion ACOs (26%). Earned shared savings rates varied by study year. High-proportion ACOs were more likely to earn shared savings in all study years, and this difference was statistically significant from 2015 to 2018 (Figure).

**Figure.**
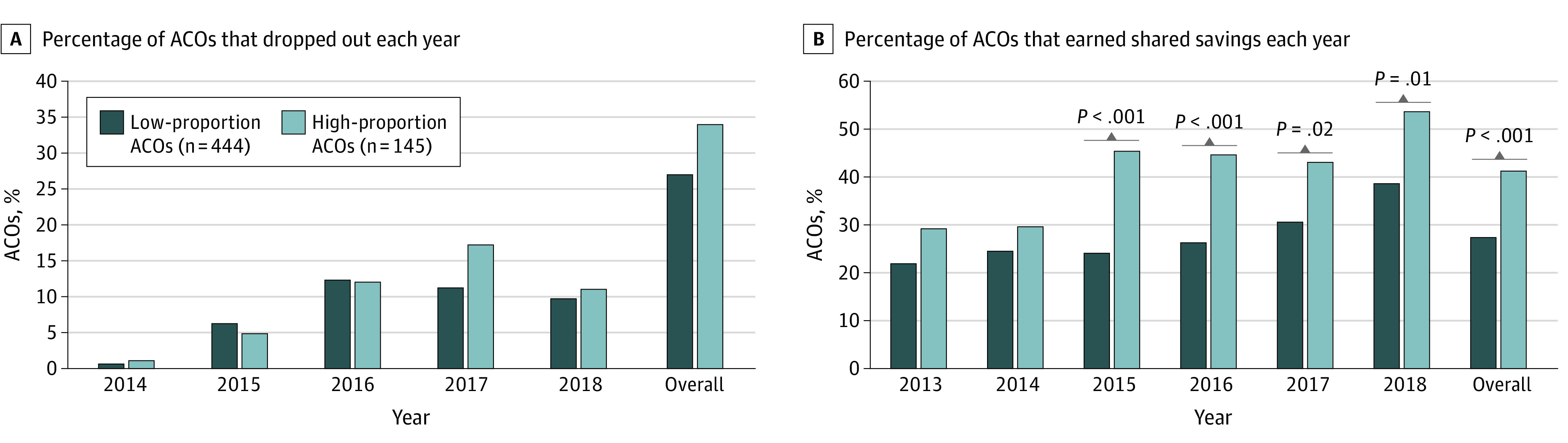
Accountable Care Organization (ACO) Shared Savings Program Exit and Earned Shared Savings Rates by Year The ACOs in the highest quartile of proportion of beneficaries of racial and ethnic minority groups were designated high-proportion ACOs, and ACOs in the lowest 3 quartiles were designated low-proportion ACOs.

Between 2012 and 2018, 168 ACOs (29%) in the study exited the SSP; 50 high-proportion ACOs (34%) exited the SSP compared with 118 low-proportion ACOs (27%) (Figure). The SSP exit rates among high-proportion ACOs increased each year, starting at 1.4% in 2014, peaking at 17.3% in 2017 and dropping to 11.2% in 2018. By comparison, low-proportion ACOs exit rates started at 0.8% in 2014, peaked at 12.3% in 2016, and dropped to 9.7% in 2018 (Figure). In a bivariate categorical analysis by year, we did not find a statistically significant difference between groups in any year.

### Logistic Regression Analysis

In unadjusted analyses, a 10–percentage point increase in the proportion of beneficiaries of racial and ethnic minority groups was associated with a 1.12-fold increase in the odds of SSP exit (95% CI, 1.00-1.25; *P* = .04; Table 2). In models adjusting for shared savings and beneficiary characteristics, the proportion of beneficiaries of racial and ethnic minority groups remained statistically significantly associated with a higher rate of SSP exit (Table 2). In models adding organizational characteristics and environmental characteristics, the proportion of beneficiaries of racial and ethnic minority groups was no longer significantly associated with the likelihood of SSP exit (Table 2). In the fully adjusted model, not earning shared savings, having a higher proportion of beneficiaries with disabilities, a higher average HCC risk score, joining the SSP in 2012 compared with the 2016 and 2017 cohorts, and having a higher proportion of primary care clinicians were also associated with exit.

**Table 2.** Logistic Regression Results, Association Between SSP Exit and Racial and Ethnic Minority Composition, Shared Savings, and Beneficiary, Organizational, and Environmental Characteristics

Characteristic	Unadjusted model, OR (95% CI)	Adjusted for earned shared savings, OR (95% CI)	Adjusted for beneficiary characteristics, OR (95% CI)	Adjusted for organizational and environmental characteristics, OR (95% CI)
Members of racial and ethnic minority groups, % (per 10 percentage points)	1.12 (1.00-1.25)	1.17 (1.04-1.31)	1.18 (1.01-1.38)	1.00 (0.78-1.29)
Earned shared savings year prior	NA	0.38 (0.24-0.60)	0.32 (0.20-0.51)	0.27 (0.14-0.51)
**Beneficiary characteristics**				
With disabilities, %	NA	NA	1.01 (0.98-1.05)	1.08 (1.02-1.13)
Dual Medicaid eligible, %	NA	NA	0.99 (0.96-1.01)	0.99 (0.96-1.03)
Percentile of average HCC risk score	NA	NA	1.02 (1.01-1.03)	1.02 (1.01-1.03)
**Organizational characteristics**				
Cohort (reference, 2012)				
2013	NA	NA	NA	1.91 (0.85-4.30)
2014				0.56 (0.26-1.23)
2015				0.77 (0.32-1.84)
2016				0.21 (0.08-0.55)
2017				0.14 (0.05-0.43)
No. of beneficiaries (per 100)	NA	NA	NA	1.01 (1.00-1.02)
Organizing entity (reference, physician led)				
Hospital led	NA	NA	NA	0.86 (0.46-1.62)
Both				0.94 (0.45-1.94)
Ever downside risk				0.68 (0.30-1.53)
No. of clinicians (per 100)				0.95 (0.91-0.99)
Primary care clinicians, %				1.02 (1.01-1.04)
Advanced practice clinicians, %				0.89 (0.86-0.91)
Out-of-network care, %				1.01 (0.99-1.03)
**Community characteristics**				
Urban	NA	NA	NA	0.59 (0.30-1.18)
Dual Medicaid/Medicare eligible, %				0.87 (0.71-1.07)
Income level less than poverty line, %				0.92 (0.83-1.01)
No. of PCPs per 1000 residents				1.82 (0.92-3.59)
County median income (per $1000)				0.96 (0.94-0.99)

Abbreviations: HCC, hierarchical condition case; NA, not applicable; OR, odds ratio; PCP, primary care physician; SSP, shared savings program.

### Marginal Effects

Using the fully adjusted model to estimate average marginal effects for low-proportion ACOs and high-proportion ACOs, we found that earing shared savings had a stronger protective effect for high-proportion than low-proportion ACOs (average marginal effect, −32 percentage points and −9.8, respectively), although the difference in effect size between the 2 groups was not statistically significant (*P* = .19). Having a higher proportion of beneficiaries with disabilities, a higher average HCC risk score, more beneficiaries, fewer clinicians, and fewer advance practice clinicians increased the likelihood of SSP exit for ACOs; again, these effect sizes was numerically greater for high-proportion than low-proportion ACOs, but the differences between them were not statistically significant (Table 3).

**Table 3.** Marginal Effects Holding Covariates at Subpopulation Means for ACOs With a High and Low Proportion of Beneficiaries of Racial and Ethnic Minority Groups

Characteristic	Marginal effects for low-proportion ACOs^a^	Marginal effects for high-proportion ACOs^a^	*P* value for interaction
Members of racial and ethnic minority groups, % (per 10 percentage points)	0.04 (– 1.80 to 1.87)	0.12 (– 5.96 to 6.20)	NA
Earned shared savings year prior	– 9.78 (– 19.26 to – 0.20)	– 32.28 (– 49.08 to – 15.4)	.19
**Beneficiary characteristics**			
With disabilities	0.54 (– 0.01 to 1.09)	1.79 (0.55 to 3.02)	.84
Dual Medicaid eligible, %	– 0.05 (– 0.31 to 0.20)	– 0.18 (– 1.01 to 0.65)	.02
Percentile of average HCC risk score for aged non–dual-eligible beneficiaries	0.12 (0 to 0.23)	0.38 (0.14 to 0.63)	.07
**Organizational characteristics**			
Cohort			
2012	1 [Reference]	1 [Reference]	NA
2013	15.03 (– 3.42 to 33.47)	16.02 (– 3.65 to 35.69)	.69
2014	– 10.62 (– 25.77 to 4.53)	– 13.4 (– 31.33 to 4.53)	.79
2015	– 5.12 (– 22.49 to 12.25)	– 6.22 (– 26.95 to 14.52)	.18
2016	– 21.91 (– 38.2 to – 5.6)	– 30.06 (– 47.41 to – 12.7)	.69
2017	– 24.19 (– 40.88 to – 7.5)	– 33.79 (– 50.97 to – 16.6)	.25
No. of beneficiaries (100s)	0.07 (– 0.05 to 0.18)	0.22 (– 0.1 to 0.55)	.58
Organizing entity			
Physician led	1 [Reference]	1 [Reference]	NA
Hospital led	– 1.06 (– 5.54 to 3.43)	– 3.67 (– 19.04 to 11.7)	.49
Both	– 0.47 (– 5.72 to 4.78)	– 1.59 (– 19.35 to 16.18)	.32
Risk model			
Always upside	1 [Reference]	1 [Reference]	NA
Ever downside risk	– 2.92 (– 9.48 to 3.65)	– 9.63 (– 29.9 to 10.65)	.55
No. of clinicians (100s)	– 0.38 (– 0.80 to 0.04)	– 1.24 (– 2.26 to – 0.20)	.07
Primary care clinicians, %	0.18 (– 0.01 to 0.37)	0.6 (0.13 to 1.08)	.89
Advanced practice clinicians, %	– 0.9 (– 1.62 to – 0.10)	– 2.98 (– 3.69 to – 2.20)	.55
Out-of-network care, %	0.06 (– 0.08 to 0.2)	0.21 (– 0.24 to 0.66)	.44
**Community characteristics**			
Geography			
Suburban	1 [Reference]	1 [Reference]	NA
Urban	– 3.91 (– 8.70 to 0.89)	– 12.9 (– 29.60 to 3.80)	.30
Percentage dual Medicaid/Medicare eligible	– 1.02 (– 2.67 to 0.62)	– 3.38 (– 8.38 to 1.62)	.01
Income level less than the poverty line, %	– 0.66 (– 1.58 to 0.26)	– 2.18 (– 4.67 to 0.31)	.89
No. of PCPs per 1000 residents	4.46 (– 1.68 to 10.60)	14.73 (– 2.03 to 31.48)	.84
County median income (1000s USD)	– 0.27(– 0.58 to 0.04)	– 0.89 (– 1.61 to −0.17)	.27

Abbreviations: ACO, accountable care organization; NA, not applicable; PCP, primary care physician.

^a^ Marginal effects calculated from fully adjusted model holding all covariates at their subpopulations means and modes. The ACOs in the highest quartile of proportion of beneficaries of racial and ethnic minority groups were designated high-proportion ACOs, and ACOs in the lowest 3 quartiles were designated low-proportion ACOs.

### Sensitivity Analyses

When we tested the sensitivity of the unadjusted analyses to single year events, we found that effect sizes and direction remained relatively consistent across all models (eTable 1 in the Supplement). Examining each race and ethnicity category individually, we found that the unadjusted bivariate association between an ACO’s racial and ethnic minority group composition and exit rate was not significant for any individual racial and ethnic minority group (eTable 2 in the Supplement). This suggests that ACOs with a more diverse population (ie, a mix of different racial and ethnic groups) were more likely to exit the SSP than ACOs that primarily served a single racial and ethnic group. When we used Research Triangle Institute–imputed values for race, the effect sizes and directions of the covariates were consistent with that of the main analyses, although the effect size for racial and ethnic minority group composition was no longer statistically significant for the unadjusted model and the model adjusted for beneficiary characteristics (eTable 3 in the Supplement). Finally, when we used a binary measure of high proportion instead of a continuous measure, the effect size of the racial and ethnic minority group variable increased for all models except the fully adjusted model, and the effect size was no longer statistically significant for the unadjusted model and the model adjusted for beneficiary characteristics (eTable 4 in the Supplement).

## Discussion

The results of this cohort study suggest that ACOs with a higher proportion of beneficaries of racial and ethnic minority groups had higher rates of SSP exit from 2012 to 2018 than ACOs with a lower proportion of beneficaries of racial and ethnic minority groups. Beneficiary characteristics, such as dual eligibility and disability, were strongly associated with this disparity, while earning shared savings had a protective effect for SSP retention.

High-proportion ACOs were more likely to earn shared savings, but also more likely to exit. While we cannot test this directly, the temporal patterns of the study findings suggest that the introduction of regional adjustments to SSP benchmarks implemented in 2017 may have made it more difficult for high-proportion ACOs to reach cost targets. Coupled with the acceleration of timelines for taking on downside risk,^16,17^ this study raises concern about retention of high-proportion ACOs in the SSP.

We also found that this effect was strongest when racial and ethnic minority group categories were considered as a group rather than individually. One explanation for this finding is that ACOs with diverse patient populations may also be located in segregated urban areas, where cost benchmarks may be more affected by regional benchmarking because of the colocation of nearby ACOs that serve more privileged White beneficiaries. Another possible explanation is that ACOs with a more diverse population face additional challenges to staying in the SSP than those with a more homogenous patient population.

The study finding that high-proportion ACOs were more likely to earn shared savings is not surprising given that the SSP was initially designed to reward improvement using an individual ACO’s historical trend. Within historical cost adjustment, ACOs may benefit from managing care for patients of racial and ethnic minority groups with high social needs whose costs can be reduced through interventions, such as transportation assistance, care management services, and social work consultation.^18^ The introduction of regional benchmarking without adequate social risk adjustment may have made it more difficult for high-proportion ACOs to earn shared savings, potentially leading to a higher rate of program exit in 2017.

This study underscores the importance of effective risk-adjustment methods that incorporate not only medical but also social risk factors to ensure that ACOs are not penalized for taking on patients with complexities,^19^ especially because a disproportionately high percentage of these patients are likely to be members of racial and ethnic minority groups.^20,21,22^ Policy changes without accompanying equity effect evaluations may substantially negatively affect racial and ethnic minority groups.^12^ In addition to assessing the equity effect of recent changes to the SSP, one supportive policy might be for the CMS to task quality improvement organizations (regional centers that provide technical support to Medicare beneficiaries and clinicians) with identifying additional strategies to support retention among ACOs with a high proportion of beneficaries of racial and ethnic minority groups. Findings could also inform the design and implementation of ACO REACH, a Medicare ACO model that aims to incorporate health equity into benchmarking and other elements of program design.

### Limitations

This study had several limitations. First, the measure of racial and ethnic minority groups combines all beneficiaries who are not White into a single category, erasing important differences between a heterogenous population. Additional work is needed to understand the nuanced experiences of different marginalized groups within ACOs and other payment reform models. Second, the generalizability of our findings is limited to SSP ACOs. However, because SSP is the biggest ACO program in the country and serves as the basis for other Medicare, commercial, and Medicaid ACO programs, we believe our findings have relevance more broadly. Third, our findings are associational and not causal. We did not examine whether ACOs that gain additional beneficaries of racial and ethnic minority groups over time are more likely to exit the SSP, although an initial analysis suggests that the racial and ethnic composition of ACOs remained relatively stable during the study period. Finally, while we hypothesize that inequitable access to Medicare SSP is associated with and exacerbates structural racism, we were unable to include explicit measures of structural racism in this analysis because of the lack of validated, theoretically derived measures.^23^ However, the application of racial labels on to human bodies could be argued as an artifact of racism itself,^24^ as race is a social construct that has no biological basis and has historically and presently been used to justify discriminatory practices and policies against groups of people.^24,25^ As such, we believe this work is a step toward understanding the contribution of payment reform to racial and ethnic inequities.

## Conclusions

In this cohort study of disparities in exit rates of SSP ACOs that serve racial and ethnic minority groups, we found that ACOs with a higher proportion of beneficaries of racial and ethnic minority groups were significantly more likely to exit the SSP program. High-proportion ACOs were also more likely to care for patients with greater disease severity and complexities. These findings suggest that an equity-centered approach to policy design and evaluation is needed to ensure that the benefits of health reform efforts and innovative care delivery models are more equitably distributed.

## Notes

Supplement. **eFigure. **Histogram of Percent Racial/Ethnic Minority Beneficiaries in an ACO’s Entry Year (n=589) **eTable 1. **Supplemental Analysis Systematically Dropping Years 2013-2017 **eTable 2. **Sensitivity Analysis 1, Logistic Regression Results Disaggregated by Individual Race/Ethnicity, Odds Ratios (95% CI) **eTable 3. **Sensitivity Analysis 2, Logistic Regression Results Using RTI Race, Odds Ratios (95% CI) **eTable 4. **Sensitivity Analysis 3, Logistic Regression Results Using Binary Variable for High Minority, Odds Ratios (95% CI)
