# Supplementary material for: Exit Rates of Accountable Care Organizations That Serve High Proportions of Beneficiaries of Racial and Ethnic Minority Groups
Source: JAMA Health Forum. 2022 Sep 30;3(9):e223398. doi: 10.1001/jamahealthforum.2022.3398 (PMC9526083; doi:10.1001/jamahealthforum.2022.3398)
Supplement: Supplement. — eFigure. Histogram of Percent Racial/Ethnic Minority Beneficiaries in an ACO’s Entry Year (n=589) eTable 1. Supplemental Analysis Systematically Dropping Years 2013-2017 eTable 2. Sensitivity Analysis 1, Logistic Regression Results Disaggregated by Individual Race/Ethnicity, Odds Ratios (95% CI) eTable 3. Sensitivity Analysis 2, Logistic Regression Results Using RTI Race, Odds Ratios (95% CI) eTable 4. Sensitivity Analysis 3, Logistic Regression Results Using Binary Variable for High Minority, Odds Ratios (95% CI) [file jamahealthforum-e223398-s001.pdf]

Supplemental Online Content

Lin SC, Maddox KEJ, Ryan AM, Moloci N, Shay A, Hollingsworth JM. Exit rates of accountable care organizations that serve high proportions of beneficiaries of racial and ethnic minority groups. *JAMA Health Forum*. 2022;3(9):e223398. doi:10.1001/jamahealthforum.2002.3398

eFigure. Histogram of Percent Racial/Ethnic Minority Beneficiaries in an ACO's Entry Year (n=589)
eTable 1. Supplemental Analysis Systematically Dropping Years 2013-2017
eTable 2. Sensitivity Analysis 1, Logistic Regression Results Disaggregated by Individual Race/Ethnicity, Odds Ratios (95% CI)
eTable 3. Sensitivity Analysis 2, Logistic Regression Results Using RTI Race, Odds Ratios (95% CI)
eTable 4. Sensitivity Analysis 3, Logistic Regression Results Using Binary Variable for High Minority, Odds Ratios (95% CI)

This supplemental material has been provided by the authors to give readers additional information about their work.

© 2022 Lin SC et al. *JAMA Health Forum.*

eFigure. Histogram of Percent Racial/Ethnic Minority Beneficiaries in an ACO's Entry Year (n=589)

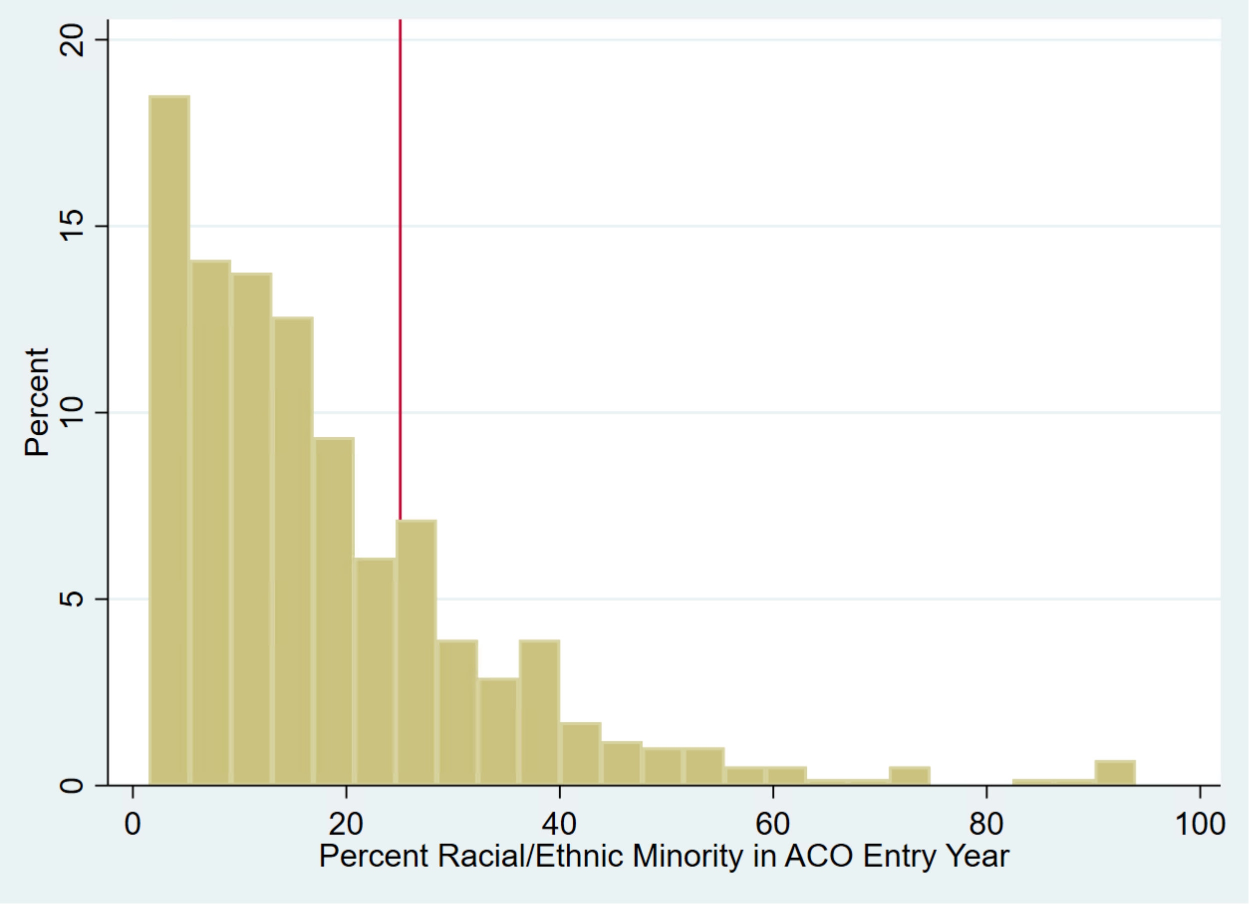

Note: Red line at 25.5% indicates the 75th percentile and the cutoff for "high minority ACOs"

© 2022 Lin SC et al. *JAMA Health Forum.*

eTable 1. Supplemental Analysis Systematically Dropping Years 2013-2017

Percent racial/ethnic minority (per 10 percentage points)	All Years (Main Model)	Dropping 2013	Dropping 2014	Dropping 2015	Dropping 2016	Dropping 2017
Odds ratio	1.12*	1.12*	1.13*	1.11	1.11	1.14*
95% Confidence Interval	[1.00,1.25]	[1.00,1.25]	[1.00,1.27	[0.98,1.26]	[0.98,1.26]	[1.01,1.30]
p-value	(0.045)	(0.052)	(0.046)	(0.095)	(0.102)	(0.041)
n	589	587	571	543	534	542

Note: odds ratios from logistic regression results, 95% confidence intervals in brackets, * p<0.05, **p<0.01, ***p<0.001

© 2022 Lin SC et al. *JAMA Health Forum.*

eTable 2. Sensitivity Analysis 1, Logistic Regression Results Disaggregated by Individual Race/Ethnicity, Odds Ratios (95% CI)

	Percent Asian Beneficiaries	Percent Black Beneficiaries	Percent Hispanic Beneficiaries	Percent Native American/ Alaskan Native Beneficiaries	Percent Other Race Beneficiaries
**UNADJUSTED MODEL**					
Percent racial/ethnic minority (per 10 percentage points)	1.30 [0.99,1.72]	1.03 [0.87,1.21]	0.97 [0.57,1.65]	0.84 [0.02,36.57]	0.83 [0.35,1.97]
**ADJUSTED MODEL**					
Percent racial/ethnic minority (per 10 percentage points)	1.92* [1.05,3.53]	0.90 [0.69,1.19]	0.54 [0.17,1.74]	3.65 [0.03,480.15]	0.44 [0.06,3.35]
Earned Shared Savings Year Prior	0.28*** [0.14,0.56]	0.31*** [0.16,0.61]	0.30*** [0.15,0.59]	0.30*** [0.15,0.60]	0.31*** [0.16,0.61]
**Beneficiary Characteristics**					
Percent disabled	1.11*** [1.05,1.19]	1.09** [1.03,1.15]	1.08** [1.03,1.14]	1.08** [1.02,1.14]	1.07* [1.00,1.14]
Percent dual Medicaid eligible	0.96 [0.92,1.01]	1 [0.97,1.03]	1.01 [0.97,1.04]	1.00 [0.97,1.03]	1.01 [0.96,1.06]
Percentile of Average HCC Risk Score for aged Non-Dual Eligible beneficiaries	1.02** [1.01,1.03]	1.02** [1.01,1.03]	1.02** [1.00,1.03]	1.02** [1.01,1.03]	1.02** [1.00,1.03]
**Organizational Characteristics**					
Cohort (Ref: 2012)					
2013	1.82 [0.81,4.08]	1.91 [0.86,4.25]	1.81 [0.81,4.04]	1.79 [0.80,3.99]	1.87 [0.84,4.14]
2014	0.49 [0.22,1.09]	0.49 [0.22,1.08]	0.5 [0.23,1.09]	0.48 [0.22,1.05]	0.48 [0.22,1.05]
2015	0.69 [0.29,1.62]	0.7 [0.30,1.65]	0.68 [0.29,1.60]	0.7 [0.29,1.64]	0.68 [0.29,1.62]
2016	0.15*** [0.05,0.45]	0.14*** [0.05,0.43]	0.14*** [0.05,0.42]	0.14*** [0.05,0.43]	0.14*** [0.05,0.41]
2017	1.00 [1.00,1.00]	1.00 [1.00,1.00]	1.00 [1.00,1.00]	1.00 [1.00,1.00]	1.00 [1.00,1.00]
Number of Beneficiaries (100s)	1.01 [1.00,1.03]	1.01 [1.00,1.03]	1.01 [1.00,1.03]	1.01 [1.00,1.03]	1.01 [1.00,1.03]
Organizing Entity (Ref: Physician)					
Hospital Led	0.86 [0.40,1.89]	0.82 [0.38,1.79]	0.84 [0.38,1.83]	0.86 [0.39,1.87]	0.84 [0.39,1.83]
Both	0.72 [0.36,1.44]	0.66 [0.33,1.31]	0.68 [0.34,1.36]	0.69 [0.35,1.37]	0.67 [0.34,1.33]
Risk Model (Ref: Always Upside)					
Ever downside risk	0.45 [0.17,1.16]	0.45 [0.18,1.17]	0.47 [0.18,1.22]	0.46 [0.18,1.18]	0.45 [0.18,1.17]
Number of Providers (100s)	0.94* [0.90,0.99]	0.94* [0.89,0.99]	0.94* [0.90,0.99]	0.94* [0.89,0.99]	0.94* [0.90,0.99]
% Primary Care Providers	1.03** [1.01,1.05]	1.03* [1.01,1.05]	1.03** [1.01,1.05]	1.03* [1.01,1.05]	1.03* [1.00,1.05]
% Advanced Practice Providers	0.89*** [0.86,0.92]	0.89*** [0.86,0.92]	0.89*** [0.86,0.92]	0.89*** [0.86,0.92]	0.89*** [0.86,0.92]
Percent Out-of-Network Care	1.01 [0.99,1.03]	1.01 [0.99,1.03]	1.01 [0.99,1.03]	1.01 [0.99,1.03]	1 [0.98,1.03]
**Community Characteristics**					
Geography (Ref: Suburban)					
Urban	0.5 [0.23,1.07]	0.57 [0.27,1.21]	0.54 [0.25,1.14]	0.54 [0.26,1.14]	0.57 [0.27,1.21]
Percent Dual Medicaid/Medicare Eligible	0.85 [0.67,1.06]	0.84 [0.67,1.06]	0.84 [0.67,1.05]	0.85 [0.68,1.06]	0.84 [0.67,1.05]
Percent Living in Poverty	0.91 [0.82,1.02]	0.92 [0.82,1.03]	0.92 [0.82,1.02]	0.92 [0.83,1.03]	0.92 [0.82,1.02]
County Median Income (Dollars)	1.00* [1.00,1.00]	1.00* [1.00,1.00]	1.00* [1.00,1.00]	1.00* [1.00,1.00]	1.00* [1.00,1.00]
Number of PCPs per 1000 Residents	2.06 [0.99,4.31]	2.13* [1.02,4.44]	2.06 [0.99,4.31]	2.14* [1.03,4.46]	2.16* [1.04,4.49]

Notes: Source: * p<0.05, **p<0.01, ***p<0.001; Hierarchical Condition Categories (HCC) score is the percentile of average HCC risk score for aged Non-Dual Eligible beneficiaries

© 2022 Lin SC et al. *JAMA Health Forum.*

eTable 3. Sensitivity Analysis 2, Logistic Regression Results Using RTI Race, Odds Ratios (95% CI)

	Unadjusted OR		Adjusted for Earned Shared Savings		Adjusted for Beneficiary Characteristics		Adjusted for Organizational, and Environmental Characteristics	
Percent Minorities (per 10 percentage points) using RTI imputed race	1.05	[0.95,1.17]	1.13*	[1.01,1.26]	1.18	[1.00,1.39]	1.15	[0.90,1.47]
Earned Shared Savings Year Prior			0.27***	[0.18,0.41]	0.22***	[0.15,0.34]	0.16***	[0.09,0.29]
**Beneficiary Characteristics**								
Percent disabled					1.01	[0.97,1.04]	1.07**	[1.02,1.13]
Percent dual Medicaid eligible					0.98	[0.96,1.01]	0.99	[0.95,1.02]
Percentile of Average HCC Risk Score for aged Non-Dual Eligible beneficiaries					1.02***	[1.02,1.03]	1.02**	[1.01,1.03]
**Organizational Characteristics**								
Cohort (Ref: 2012)								
2013							1.77	[0.79,3.99]
2014							0.51	[0.23,1.13]
2015							0.68	[0.29,1.61]
2016							0.17***	[0.06,0.46]
2017							0.09***	[0.03,0.28]
Number of Beneficiaries (100s)							1.01	[1.00,1.03]
Organizing Entity (Ref: Physician)								
Hospital Led							0.86	[0.45,1.64]
Both							0.87	[0.41,1.83]
Risk Model (Ref: Always upside)								
Ever downside risk							0.88	[0.38,2.02]
Number of Providers (100s)							0.95*	[0.91,0.99]
% Primary Care Providers							1.02*	[1.00,1.04]
% Advanced Practice Providers							0.89***	[0.86,0.92]
Percent Out-of-Network Care							1.01	[0.99,1.03]
**Environmental Characteristics**								
Geography (Ref: Suburban)								
Urban							0.67	[0.33,1.39]
Percent Dual Medicaid/Medicare Eligible							0.86	[0.69,1.07]
Percent Living in Poverty							0.89*	[0.80,0.99]
Number of PCPs per 1000 Residents							1.85	[0.90,3.81]
County Median Income ($1000)							0.96**	[0.93,0.99]

Notes: * p<0.05, **p<0.01, ***p<0.001; Hierarchical Condition Categories (HCC) score is the percentile of average HCC risk score for aged Non-Dual Eligible beneficiaries

© 2022 Lin SC et al. *JAMA Health Forum.*

eTable 4. Sensitivity Analysis 3, Logistic Regression Results Using Binary Variable for High Minority, Odds Ratios (95% CI)

	Unadjusted OR	Adjusted for Earned Shared Savings	Adjusted for Beneficiary Characteristics	Adjusted for Organizational, and Environmental Characteristics
High Minority (Top Quartile of Percent Minority Beneficiaries)	1.45 [0.97,2.17]	1.67* [1.10,2.53]	1.41 [0.88,2.25]	0.97 [0.49,1.95]
Earned Shared Savings Year Prior		0.38*** [0.24,0.60]	0.32*** [0.20,0.52]	0.27*** [0.14,0.51]
**Beneficiary Characteristics**				
Percent disabled		1.01 [0.98,1.05]	1.08** [1.02,1.13]	1.01 [0.98,1.05]
Percent dual Medicaid eligible		0.99 [0.97,1.01]	0.99 [0.97,1.02]	0.99 [0.97,1.01]
Percentile of Average HCC Risk Score for aged Non-Dual Eligible beneficiaries		1.02*** [1.01,1.03]	1.02** [1.01,1.03]	1.02*** [1.01,1.03]
**Organizational Characteristics**				
Cohort (Ref: 2012)				
2013				1.89 [0.85,4.20]
2014				0.56 [0.26,1.20]
2015				0.76 [0.33,1.78]
2016				0.20** [0.08,0.53]
2017				0.14*** [0.05,0.42]
Number of Beneficiaries (100s)				1.01 [1.00,1.02]
Organizing Entity (Ref: Physician)				
Hospital Led				0.86 [0.46,1.61]
Both				0.94 [0.45,1.93]
Risk Model (Ref: Always upside)				
Ever downside risk				0.68 [0.30,1.53]
Number of Providers (100s)				0.95* [0.91,0.99]
% Primary Care Providers				1.02* [1.01,1.04]
% Advanced Practice Providers				0.89*** [0.86,0.91]
Percent Out-of-Network Care				1.01 [0.99,1.03]
**Community Characteristics**				
Geography (Ref: Suburban)				
Urban				0.6 [0.30,1.18]
Percent Dual Medicaid/Medicare Eligible				0.87 [0.71,1.07]
Percent Living in Poverty				0.92 [0.83,1.01]
Number of PCPs per 1000 Residents				1.81 [0.92,3.59]
County Median Income (Dollars)				0.96* [0.94,0.99]
N	589			

Notes: * $p<0.05$, ** $p<0.01$, *** $p<0.001$; Hierarchical Condition Categories (HCC) score is the percentile of average HCC risk score for aged Non-Dual Eligible beneficiaries

© 2022 Lin SC et al. *JAMA Health Forum.*
